# Investigating the Internal Deterioration of the Auriga Statue of Mozia Island, Sicily, through Ultrasonic and Ground-Penetrating Radar Studies †

**DOI:** 10.3390/s24196450

**Published:** 2024-10-05

**Authors:** Patrizia Capizzi, Raffaele Martorana, Alessandra Carollo

**Affiliations:** Department of Earth and Marine Sciences, University of Palermo, 90123 Palermo, Italy; patrizia.capizzi@unipa.it (P.C.); alessandra.carollo02@unipa.it (A.C.)

**Keywords:** ultrasonic tomography, UST, ground-penetrating radar, GPR, heritage, Greek statue, Auriga, Mozia

## Abstract

The Greek marble statue of the Auriga of Mozia Island, in Sicily, is the most important artwork displayed at the Whitaker Foundation Archaeological Museum. It underwent geophysical investigations twice, in 2012 and 2021, to assess the marble’s degradation. The 2012 investigation prepared the statue for transfer to the Paul Getty Museum in New York and repositioning on an anti-seismic pedestal. The 2021 investigation evaluated potential new damage before another transfer. Both investigations utilized 3D ultrasonic tomography (UST) to detect degraded marble areas and ground-penetrating radar (GPR) to identify internal discontinuities, such as fractures or lesions, and locate metal pins that were previously inserted to reassemble the statue and its pedestal. Results from the UST indicate an average marble velocity of approximately 4700 m/s, suggesting good mechanical strength, with some areas showing lower velocities (~3000 m/s) within the material’s variability range. The GPR profiles demonstrated internal signal homogeneity, excluding internal fracture surfaces or lesions, and confirmed the presence of metallic pins. This study highlights the effectiveness of integrating UST and GPR for non-invasive diagnostics of marble sculptures, providing detailed insights into the marble’s condition and identifying hidden defects or damage.

## 1. Introduction

**Figure 1:**
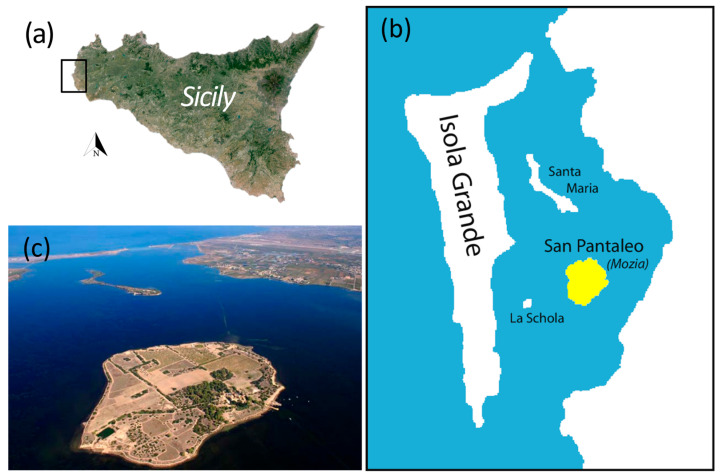
Geographical map of Sicily (**a**), where the black rectangle indicates the coastal lagoon of Stagnone (**b**), in the center of which is the island of Mozia (**c**).

Mozia is a significant archeological site located between the municipalities of Trapani and Marsala in western Sicily (Figure 1a). The site encompasses the island of San Pantaleo, also referred to as Mozia Island (Figure 1b), which is situated in a notable shallow lagoon known as the “Stagnone” (Figure 1c).

Mozia was an important colony of Carthage, the Phoenician-founded city in north Africa. Over the past few decades, extensive archeological campaigns across the island of Mozia, often supported by geophysical surveys, have uncovered significant remains of the ancient Phoenician settlement [1]. These investigations have provided invaluable insights into the urban planning [2], architectural styles, and daily life of the Punic civilization that once thrived on the island. The integration of advanced geophysical techniques has allowed archeologists to detect and map subsurface structures, leading to more targeted and effective excavations. As a result, the collective findings from these efforts have enriched our understanding of the historical and cultural heritage of Mozia.

The Carthaginians played a fundamental role in the ancient history of Sicily, engaging in the construction, conquest, and loss of settlements in conflict with various Greek colonies on the island [3]. However, few archeological remains have survived in Sicily to testify to the presence of the Phoenician culture on the island. Mozia is therefore the most important of these due to the abundance and richness of the artifacts discovered.

When Mozia was destroyed by the Syracusans in the fourth century BCE [4], the survivors founded the city of Lilybaeum (the modern Marsala) on a more defensible headland along the nearby Sicilian coast [5,6].

Over time, the long external island (Isola Grande) that protects Mozia from the open sea caused sediment accumulation that transformed the waters surrounding Mozia into a shallow lagoon. Here, several ancient shipwrecks have been discovered: some of these are Phoenician warships refuging at Lilybaeum after the naval Battle of the Egadi Islands in 241 BC, in which the Phoenician were defeated by Romans. The shipwreck found in the best conditions is currently exhibited in the archeological museum in Marsala [7].

In past centuries, the salt-extraction industry along the mainland coast was thriving, and it continues to this day. However, the memory of Mozia had faded over time until 1902, when Joseph Whitaker, an English exporter of Marsala wine, purchased the island and began excavations that led to the rediscovery and enhancement of the archeological site. Whitaker also founded a museum on the island, which is still managed by the foundation that bears his name.

**Figure 2:**
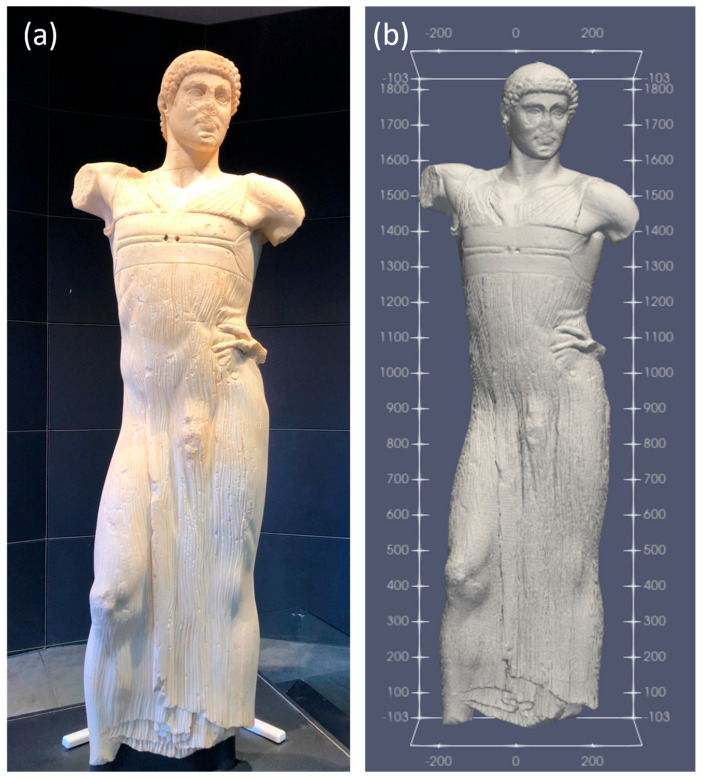
(**a**) Frontal view of the statue of the “Auriga” (charioteer), housed at the Whitaker Museum of Mozia; (**b**) a 3D digital reconstruction of the statue’s surface, used for the correct positioning of sensors and for the graphical rendering of the tomographies.

The highlight of the museum is the “Auriga” (charioteer) statue (Figure 2a), also known as the Youth of Mozia [8], a masterpiece of classical marble statuary, dating back to the mid-5th century BCE. It was discovered on 26 October 1979, by Gioacchino Falsone [9] during a series of excavations conducted by archeologists from the University of Palermo, which began in 1977 on the Mozia island. The statue was found under a pile of debris in the ancient industrial area. The life-size statue was made with marble from Asia Minor. Its origin, artistic style, and even symbolic representation are shrouded in mystery. The figure depicts a male youth (an ephebe) draped in fabric, possibly an ancient Greek charioteer. It was likely brought to the island of Mozia by the Carthaginians after plundering Selinunte in 409 BCE. This masterpiece must have been created by an important Greek artist of the first half of the 5th century BCE, but neither the identity of the young man nor the place where the statue was originally to be exhibited on the island are known. When the statue was discovered, its head was detached from the body but juxtaposed to it, a sign that the fracture was due to ground pressure. The absence of limbs, metallic accessories, and the base clearly indicate that the statue was not in situ. It was most likely intended to be erected in the nearby sanctuary, from where it would have been dragged to the discovery site after being toppled during the siege by the Syracusans.

This research originated from the need, on two separate occasions in the exhibition history of the statue, to perform non-invasive diagnostic investigations on the statue to assess its potential movement. This is a delicate task, considering the historical and artistic value of this remarkable artifact and the necessity to execute measurements in the hall of the archeological museum of the Giuseppe Whitaker Foundation, where the statue is displayed.

The initial investigations date back to 2012, when the decision was made to exhibit the statue in the Getty Museum of Malibu (Los Angeles, CA, USA). On this occasion, specific studies were conducted for the design and construction of an anti-seismic base for the artwork, aiming to minimize the seismic risk not only during its residency in Los Angeles but also for its subsequent return to its usual location at the Whitaker Museum in Mozia.

For this occasion, it would have been necessary to dismantle the previously installed pedestal supporting the statue, which had been mounted using two steel pins inserted at the base of the statue. The studies, carried out in collaboration between the University of Palermo and the Restoration Center of the Sicilian region, included, among other investigations, a 3D ultrasonic tomography (UST) and ground-penetrating radar (GPR) surveys, both performed on the lower part of the statue in order to identify the exact position of the metal pins and to study the degradation state of the marble, also due to a suspected lesion in that lower zone of the statue.

The second opportunity arose at the end of 2021, when the idea of creating an exhibition for the statue in the metope hall of the Archaeological Museum “A. Salinas” in Palermo was proposed. The University of Palermo was thus tasked with conducting non-invasive examinations to evaluate the marble’s state of degradation and the presence of possible lesions that could compromise its integrity during relocation.

Again, on this occasion, some high-resolution GPR profiles and a 3D UST were performed, but this time on the entire volume of the statue.

On both occasions, a precise 3D survey of the statue was used, aimed at the correct location of the ultrasonic measurement points and GPR profiles and to obtain a 3D rendering of the tomographic models (Figure 2b).

## 2. Materials and Methods

In the field of cultural heritage diagnostics, non-destructive techniques serve as valuable tools for identifying defects such as fractures, areas of degradation, or junction zones within artifacts [10,11,12]. Among these techniques, UST and GPR are frequently chosen due to their non-invasive nature and rapid execution. The application of UST allows for the detection of internal flaws by analyzing the propagation of sound waves through the material. When independently used, UST is highly effective in identifying cracks, voids, and other structural defects within marble [10,13]. It also can evaluate the uniformity of the marble, identifying areas of varying density [14,15]. In fact, cracks or deteriorations cause significant reductions in wave velocity compared to that of a homogeneous material [16,17]. On the other hand, GPR utilizes electromagnetic waves to create subsurface images, revealing hidden structures and anomalies. The use of the GPR method allows for a greater depth of penetration compared to US tomography, managing to identify any structures and discontinuities that are not detectable with US tomography alone. Furthermore, GPR can identify materials that are different in terms of their electrical permittivity or electrical conductivity, but which have similar ultrasonic velocities and are therefore difficult to distinguish with US tomography alone. Finally, GPR allows for faster data acquisition compared to US tomography, allowing for the investigation of larger surfaces in less time [10].

In this work, we used a combined approach of these advanced diagnostic methods, even from a multi-scale perspective, to provide a holistic assessment of the marble, combining detailed surface information with deep structural insights. This aims to improve the diagnostic accuracy, enabling more precise localization and characterization of defects.

### 2.1. Ultrasonic Tomography

Ultrasound refers to elastic vibrations whose frequency range extends from values above 20 kHz up to over 200 MHz. Ultrasonic waves are generated by exploiting the piezoelectric properties of certain materials; these properties involve the ability of these materials to contract and expand when subjected to an alternating electric field. If the alternating electric field has the appropriate frequency, the material’s vibrations produce elastic waves of ultrasonic frequency. Unlike sonic oscillations, ultrasonic waves do not transmit as easily through gasses, such as air; instead, they can travel long distances while remaining practically unchanged if the medium they travel through is a homogeneous liquid or solid. In the presence of discontinuities, such as different materials, these waves are reflected and refracted.

Ultrasonic waves can be either pressure or shear waves and have a penetration capacity into the material that is inversely proportional to their frequency. Naturally, the penetration capacity also depends on the intrinsic characteristics of the material being traversed.

Ultrasonic tomography (UST) is a powerful non-destructive testing technique that was originally developed in the medical field to investigate the internal structure of human tissues. Its versatility has made it a valuable tool in other fields, such as civil engineering and diagnostics of cultural heritage. In fact, this technique is particularly useful for characterizing mechanical stress and locating discontinuities within stone and wooden materials. This method provides information on physical parameters such as the velocity of elastic waves and the elastic modulus that allow us to gather evidence of invisible anomalies related to areas of decay and structural weaknesses hidden within the investigated object, such as cracks, voids, inclusions, or defects, based on the mechanical properties. It is also possible to assess the extent of visible decay on the surface and to measure the depth and extent of fractures. The imaging algorithms used exploit a linear approximation of the direct problem (Born approximation) and assume that a transverse section of the investigated object is isotropic. In the field of cultural heritage, UST has often been successfully used [18,19,20,21,22,23] thanks to its high sensitivity and resolution and at the same time acceptable penetration power. Specifically, ultrasonic tomography has been successfully used to investigate the degradation state of marble in numerous artifacts of artistic significance [14,24,25,26,27]. This allows us to locate relatively small discontinuities, provided that they are larger than the wavelength used. At the same time, there are several specific challenges. Ultrasonic waves often do not offer sufficient material contrast due to the relatively small variations in elastic waves velocity. This can make it difficult to distinguish between different types of materials or identify small defects. Cultural heritage objects like statues can be extremely complex, with intricate designs and a mixture of different materials. This can make it difficult to interpret the results of ultrasonic tests [24]. Moreover, although UST is a non-destructive technique, there is always a risk of damage when applying any testing method to delicate and irreplaceable artistic objects. Finally, interpreting the results of UST requires a high level of expertise. Misinterpretation of results can lead to incorrect conclusions about the state of conservation of a heritage object.

#### Ultrasonic Data Acquisition

Ultrasonic inspection for the detection of internal discontinuities in materials is carried out with two basic techniques: the so-called “transparency” and “reflection” techniques. Of these, we preferred the transparency technique, in which two transducers are used, generally positioned opposite to each other on the opposite surfaces of the medium to be examined; one of the two transducers acts as an emitter, the other as a receiver.

Prior to the execution of the tomography measurements, it is good practice to cover the statue with a transparent polyvinyl chloride film to preserve its surface from possible interactions with the gel used for the ultrasonic measurements. Although the presence of the ultra-thin film on the surface may somewhat alter the spectral shape of the signal generated by the ultrasonic source, it should not significantly affect the arrival times of the ultrasonic waves, on which the US tomography measurements are exclusively based.

The data were acquired using TDAS 16 instrumentation produced by Boviar. This is a multichannel device (16 channels) that allows us to acquire, through an electronic switch, four channels at a time with a maximum sampling frequency of 1.25 MHz. The receiving and transmitting probes have a central frequency of 55 kHz; for precise measurements, as in this case, the probes are equipped with special aluminum supports in the shape of a cone that allow for a more accurate positioning of the sensor on the artifact.

The piezoelectric transmitter TSG-55 is of the “sandwich” type with pre-loaded ceramics and allows us to generate pulses with a high signal transmission power, with a frequency centered on 55 kHz. The piezoelectric accelerometric receivers, RSG-55, were designed to have a high sensitivity in a frequency range of the received signals that goes from 1 kHz to 8 kHz, with a peak at 6 kHz (30 V/g), which is characteristic for investigations of structures made of not particularly fast materials (historic buildings, degraded and/or fractured rocky materials, etc.) and a good and relatively stable (linear response) stability for frequency ranges from 10 kHz to 70 kHz, which are typically used for investigations on concrete or rock samples, both for in situ tests and in the laboratory.

The data acquisition software, managed by a notebook, in addition to allowing for the setting of the main acquisition parameters (temporal range of 0.1 ms^−1^ s, sampling frequency, and gains) allows for the control of the quality of the signals and the estimation of the arrival times through the real-time visualization of the waveforms; the good quality of the data was ensured by the possibility of summing and averaging hundreds of signals until reaching very high stacking values.

**Figure 3:**
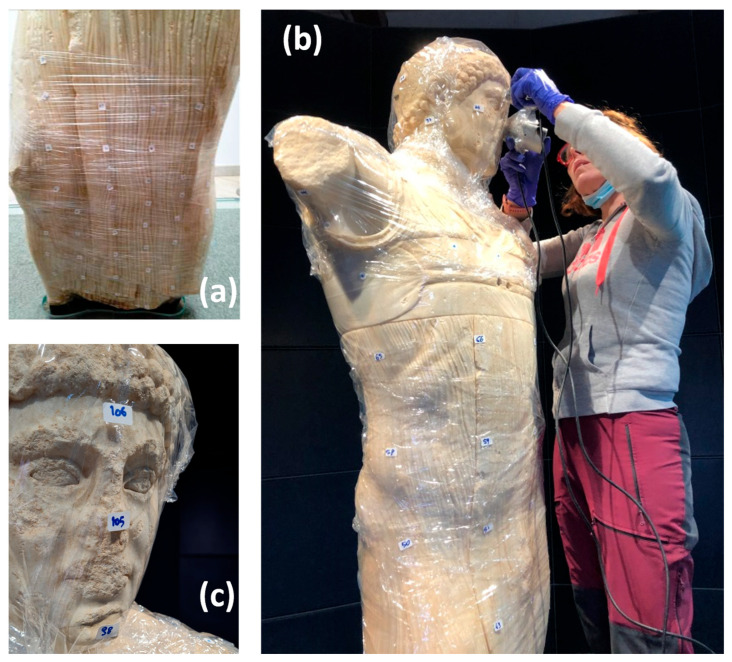
Various stages of UST data acquisition on the statue of the Auriga of Mozia covered with transparent film: (**a**) view of adhesive paper circles during the 2012 measurements; (**b**) US measurements in 2021; (**c**) some measurement points on the head of the statue in 2021.

During the 2012 test, only the lower part of the statue was investigated (Figure 3a), from the base up to a height of 60 cm. This is the zone of a possible injury, hypothesized based on the presence of a surface clay vein. The aim was also to find the exact location and length of the metallic pins that secured the statue to its pedestal. Here, 96 measurement points were selected, the positions of which were chosen to favor good coupling between the transducers and the surface of the statue, as well as to obtain a sampling density as homogeneous as possible (Figure 3a).

In total, 1060 signals were acquired and processed, on which the first-arrival times of the elastic waves were picked.

The distance between the measurement points varied between approximately 7 and 10 cm. For the detection of the exact position of the measurement points, adhesive circles were used, which were photographed to accurately locate their position on the 3D relief of the statue (Figure 2b).

In 2021, 114 measurement points were used instead, distributed homogeneously over the entire surface of the statue (Figure 3b). In this case too, the measurement points were marked with adhesive labels to accurately locate their position on the 3D relief of the statue (Figure 3c). In this case, 371 signals related to as many kinematic paths were acquired and processed.

### 2.2. Ground-Penetrating Radar

Ground-penetrating radar (GPR) is a specific type of radar that uses electromagnetic waves with frequencies between 10 MHz and 3 GHz to detect structures and targets underground or within materials [28,29]. Due to its rapid data acquisition and high-resolution results, this method is one of the most widely used non-destructive testing (NDT) techniques for diagnosing the degradation state of buildings, monuments, and other ancient artifacts [30,31,32]. It can identify voids and moisture, fractures and detachments, as well as metallic inserts.

GPR exploits the physical phenomena such as reflection, refraction, and diffraction that an electromagnetic wave undergoes when encountering discontinuities within the investigated medium. This is caused by variations in the electrical and magnetic properties of the materials traversed, primarily electrical permittivity but also magnetic permeability and electrical conductivity [33]. These variations can be due to changes in the chemical composition of the investigated material, as well as fractures [34], voids [35], humidity [36], or metallic pins in the artifact. Due to the aforementioned characteristics, GPR has recently proven useful for investigating the presence of discontinuities within marble blocks or artifacts [15,37,38,39,40].

A GPR system generally consists of a control unit connected to an antenna system, which sends electromagnetic pulses and captures the reflected and/or refracted signals. The electromagnetic signals are digitized, displayed on a PC, and saved on a mass storage device [28]. The electromagnetic pulses emitted by the transmitting antenna can be reflected or refracted by dielectric or magnetic discontinuities and captured by a receiving antenna. The time elapsed between the pulse emission and the reception of the reflected or transmitted signal provides information on the depth of the detected discontinuity, while a velocity analysis allows for the estimation of the dielectric and magnetic properties of the traversed material.

GPR data can be acquired by various techniques. The most widely used is the reflection method, where the transmitting and receiving antennas move together along the investigated surface. The acquired data are then displayed in the form of a section, with the reflection time along the vertical axis and the antenna position along the horizontal axis.

#### GPR Data Acquisition

GPR investigations were carried out using the Aladdin georadar system from IDS (Ingegneria Dei Sistemi, 2006, Naples, Italy), equipped with two pairs of 2 GHz antennas contained in a small box, in order to obtain a high level of detail of the acquired data. The profiles were created by configuring the antennas in bipolar mode, that is, using two pairs of antennas perpendicular to each other. This mode allows for data to be acquired with two different positions of the dipoles with respect to the direction of advancement: dipoles parallel or perpendicular to the acquisition direction.

**Figure 4:**
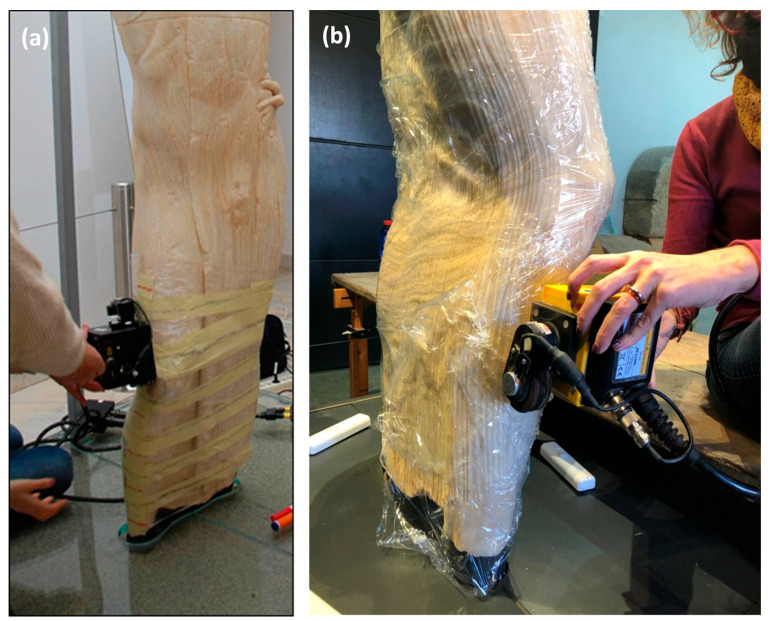
GPR data acquisition on the statue of the Auriga (charioteer) of Mozia: (**a**) measurements taken in 2012 along horizontal closed paths marked with white adhesive tape; (**b**) a moment of the acquisition in 2021 along the vertical direction.

The 2012 GPR investigations focused only on the lower part of the statue, where it was assumed that there were lesions, and specifically, the first 60 cm of height was investigated (Figure 4a). The investigated part was preliminarily covered with a polyvinyl chloride film. Twelve profiles were executed perpendicular to the longitudinal axis of the statue, i.e., along roughly ellipsoidal traces (Figure 4a), with a distance of 5 cm between them. The traces of the profiles were marked with adhesive paper tape. The length of the twelve profiles varied from a minimum of 96 cm to a maximum of 107 cm, according to the perimeter variations of the investigated surface.

In 2021, it was decided to carry out 30 GPR profiles of varying lengths and different positions, with directions sometimes horizontal and sometimes vertical, in order to cover the entire statue in a fairly homogeneous manner (Figure 4b).

Table 1 lists, for each profile, the direction and position relative to the statue.

On both occasions, the acquisition parameters included a Butterworth-type band-pass frequency filter, horizontal stacking, and Range Gain to define the law of gain variation as a function of time and to compensate for the attenuation of the background geometric spreading. Furthermore, a temporal acquisition range of 5 ns was used. This value was obtained by estimating the average speed of electromagnetic waves in the medium, equal to about 0.084 m/ns, thanks to the slopes of the hyperbola branches that were present in the data and from calibration tests carried out on the statue. Considering this value, the maximum theoretically achievable investigation depth is about 18 cm.

Each GPR profile was processed to eliminate the coherent and incoherent noise present in the original data. First, static correction was applied to correct the time delay in each trace. Subsequently, background removal was applied to all profiles in order to eliminate constant noises in the space dimension. Finally, Kirchoff migration was performed based on a constant propagation speed to bring the reflections and diffractions back to the correct position of the object that generated them [41].

## 3. Results

The results of the UST and GPR investigations on the statue of the Auriga were interpreted, and a comparative analysis was conducted between the two techniques, as well as between two temporal phases, separated by nine years, to highlight any differences. This comparative study aims to identify changes in the statue’s condition over time, providing insights into the effectiveness of conservation efforts and the progression of any deterioration.

### 3.1. UST Results

**Figure 5:**
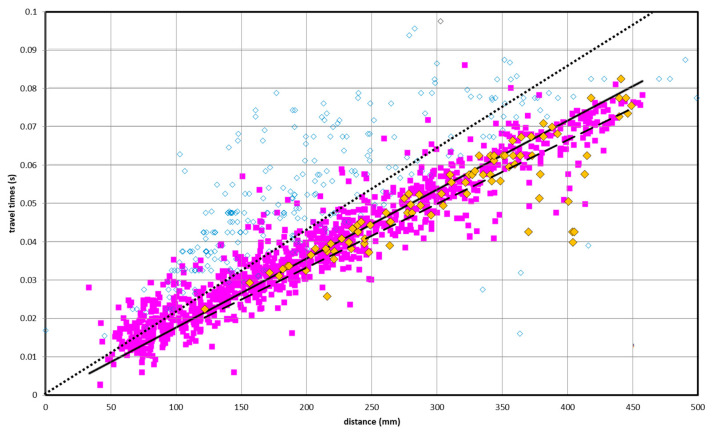
Measured ultrasonic travel times versus source–receiver distance. The 2012 data (in magenta), acquired only in the lower part of the statue, are compared with those acquired in 2021 on the whole surface of the statue (white diamonds) and with those, among these, acquired in the lower part of the statue (yellow diamonds). The solid line represents the linear regression of the 2012 data, while the dotted and dashed lines represent, respectively, the linear regression of the entire 2021 dataset and of only the data acquired in the lower part of the statue.

Figure 5 shows the comparison of the measured ultrasonic travel times of 2012 (in magenta), acquired only in the lower part of the statue (first 60 cm in height), those measured in 2021 on the whole surface of the statue (white diamonds), and those, among these, that were acquired in the lower part of the statue (yellow diamonds). Essentially, the data acquired in the same zone, over time, show similar distributions of apparent velocities and regression lines (solid and dashed in Figure 5) that are very close to each other. However, the linear regression related to the whole 2021 dataset (dotted line in Figure 5) shows a lower average velocity. This can be attributed to the presence of the metal inserts at the base of the statue, which increase the velocity values compared to the rest of the statue. Some anomalous arrival time values among the 2021 measurements were considered to be caused by the weight of imprecise picking of noisy signals recorded in zones of the statue that have a very articulated surface. For this reason, these data were considered outliers and were excluded from the tomographic inversion process.

**Figure 6:**
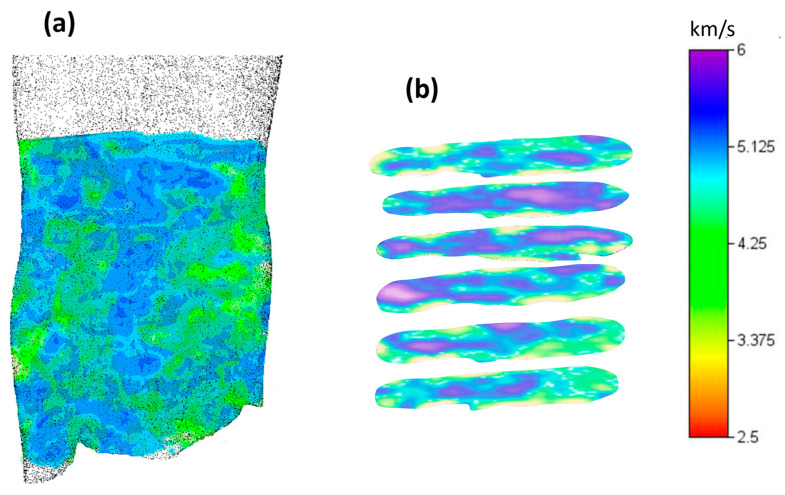
Results of the UST performed in 2012 on the statue of the Auriga (charioteer) of Mozia: (**a**) a 3D visualization of the tomographic model and (**b**) horizontal sections of the tomographic model.

The 3D mesh used for the inversion of the 2012 ultrasonic data consisted of cubic cells with a side length of 5 m. The inverse model (Figure 6a) showed fairly heterogeneous velocity values, ranging from a minimum of approximately 4000 m/s and a maximum of approximately 7000 m/s. Low values indicate a suboptimal condition of the marble, especially in correspondence with localized anomalies, likely corresponding to poor mechanical characteristics.

In Figure 6b, six sections that are approximately 7 cm equidistant are shown. In these sections, the distribution of velocities transversely to the vertical direction can be better observed. In particular, a low-velocity zone characterizes the right end of the statue in its first 25–30 cm from the bottom. This zone has velocities lower than 5000 m/s. Therefore, it is a comparatively degraded volume. Other low-velocity zones are visible in the central and left external zones, where the pleating of the garment may, however, generate low-velocity artifacts in the inverse model. Also, any voids or cavities that could be present in the lower part of the statue, not visible and not documented by the survey, could give rise to such artifacts.

The results of the 2021 UST show an average marble velocity of about 4700 m/s, indicating a good mechanical strength of the material. There are widespread areas with lower velocities (around 3000 m/s), which still fall within the material’s variability range. A comparison was made with the ultrasonic data acquired in January 2012 during a previous diagnostic campaign.

**Figure 7:**
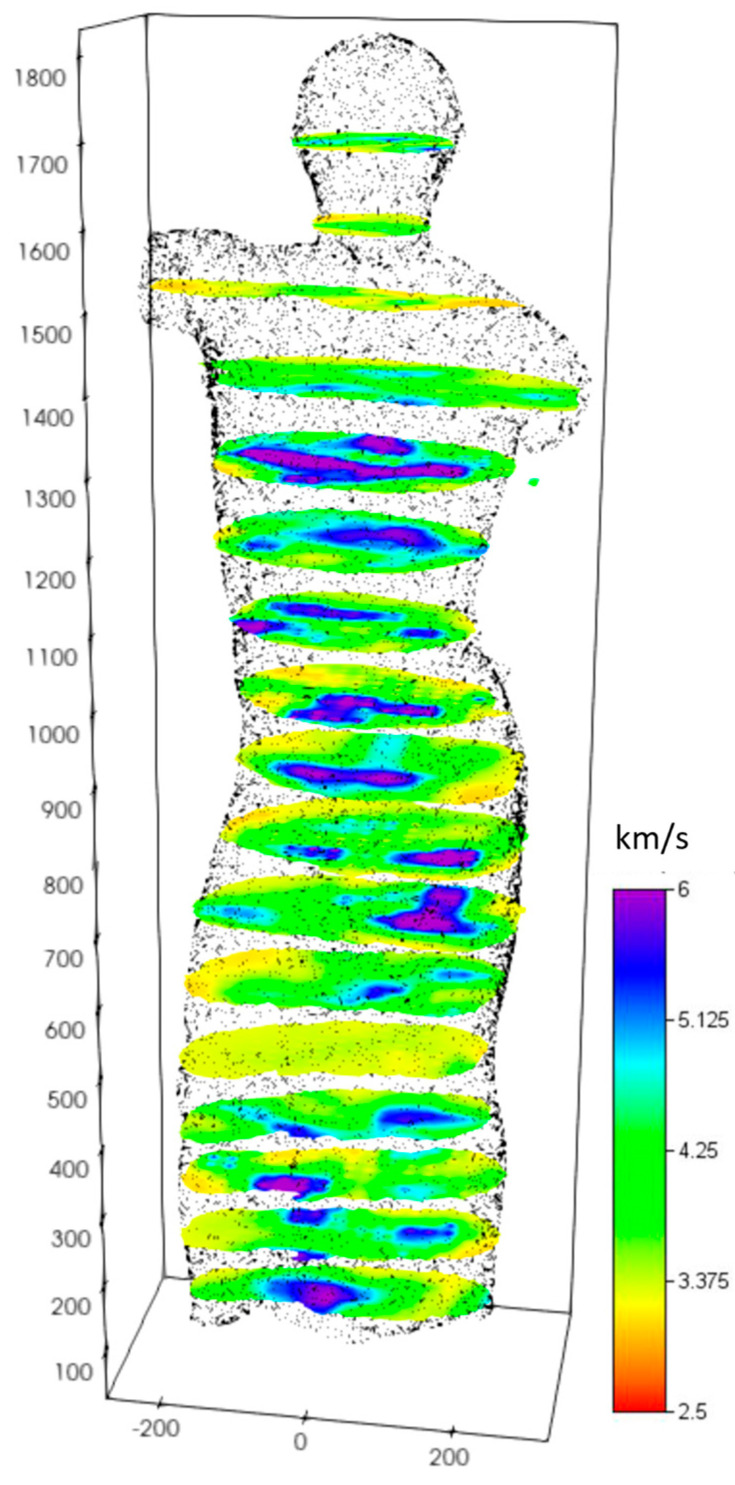
Results of the UST performed in 2021 on the statue of the Auriga (charioteer) of Mozia. Seventeen horizontal sections, spaced ten centimeters apart, are shown.

Seventeen sections that are approximately equidistant from each other by 10 cm are shown in Figure 7, where it can be observed that generally, the outermost zones are characterized by lower velocities (still above 3000 m/s), possibly due to the marble’s processing. These values were also found to be in correspondence with the clay vein in the lower right leg, as detected in the investigations carried out in 2012. In this case as well, there is no clear demarcation line suggesting an internal lesion, which would presumably show significantly lower values than those detected. It is therefore confirmed that the outermost areas of the statue show values lower than the average measured velocities, consistent with the marble’s processing, but no detectable internal lesions are evident.

**Figure 8:**
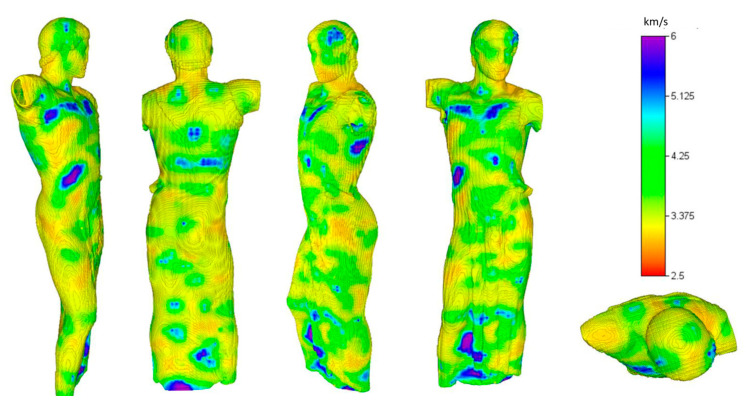
Three-dimensional rendering of the UST model from data acquired in 2021 on the Auriga (charioteer) of Mozia.

In Figure 8, the same results are represented in a three-dimensional rendering from different viewpoints. This representation highlights the velocities of the marble areas near the statue’s surface, noting that there are some zones of weakness where the velocity is less than 3500 m/s, particularly in the neck and shoulder areas, as well as the hips.

### 3.2. GPR Results

**Figure 9:**
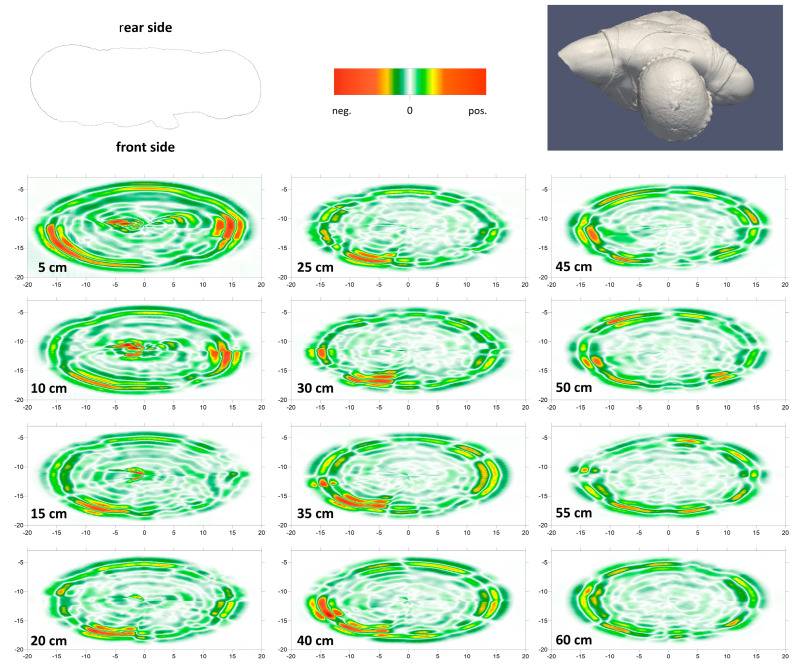
GPR sections related to the data acquired in 2012, in the horizontal direction, on approximately elliptical circuits with an interdistance of 5 cm. The height of each section from the base (in centimeters) is written in each section. The top part of the sections refers to the rear side of the statue.

The main purpose of the GPR analyses carried out in 2012 was to locate and measure the pins that supported the statue at that time before its temporary transfer to the Paul Getty Museum and subsequent relocation to the Whitaker Museum on a new anti-seismic base. The results of the GPR investigations carried out in 2012 are shown in Figure 9. For each of the twelve perimeter profiles executed, marked on the statue with adhesive tape (Figure 4a), the elliptical sections are presented (Figure 9), obtained by operating with electromagnetic dipoles perpendicular to the direction of advancement. In the first four sections, the metal pins are recognizable, and their length of about 18–20 cm can be deduced. However, their location is less clear due to the complex geometry of the real section boundaries, which are not perfectly elliptical and could not be reconstructed via software in the GPR sections. A pacometer survey allowed for more precise identification of the pins’ locations, slightly closer to the rear side of the statue and approximately 30 cm apart from each other.

In 2021, the GPR profiles were acquired on the most coplanar alignments, and no topographic correction was applied to the data. In fact, it was considered that the topographical correction was not necessary for the purposes of the investigations. The rock/air surface was identified in the profiles, and the homogeneity of the crossed part of the statue was analyzed.

**Figure 10:**
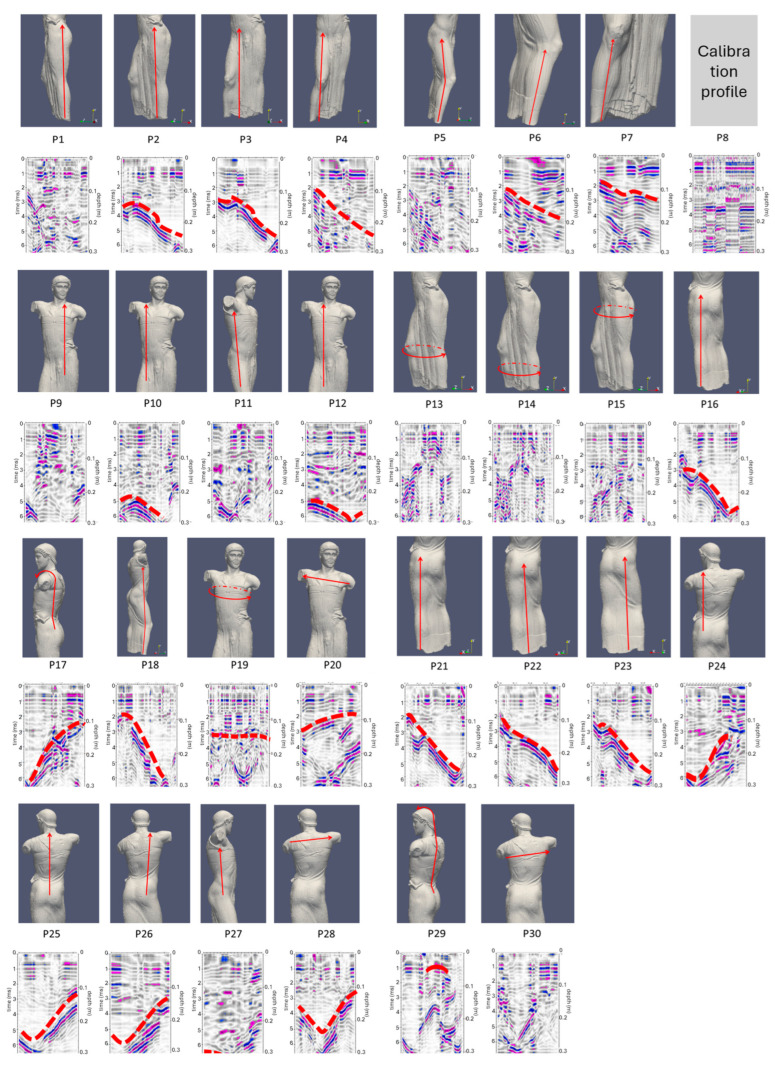
GPR profile collected in 2021 on the Auriga (charioteer) of Mozia. The reflection amplitudes are represented in a color scale from blue (maximum negative amplitude) to magenta (maximum positive amplitude). The red arrows indicate the traces of each profile on the surface of the statue. The dashed red lines highlight, in each GPR section, the reflection caused by the marble/air contact, opposite to the acquisition surface.

All profiles acquired in 2021 on the Auriga are shown in Figure 10. For each profile, the trace on the surface of the statue is displayed. In the section, where visible, the reflection caused by the marble/air contact surface, opposite to the direction in which the profile was acquired, is indicated with a dashed red line.

The profiles were created out to highlight any internal discontinuity surfaces, which can be interpreted as the presence of fractures and/or lesions. It should be noted that there are no relevant anomalies in the internal part of the statue that can be attributed to discontinuities or alterations in the material. Only a few anomalies are highlighted due to the presence of internal metal pins due to previous restorations (P29). The pins, already highlighted in previous diagnostic investigations carried out in 2012, were not investigated but are evident, such as those at the base in the circular profile P14 and the neck pin in profile P29, where its reflection hyperbola has been highlighted in red. This latter pin is not evident in profile P28, confirming its reduced dimensions, as already reported in the 2012 investigations. In all the acquired ground-penetrating radar profiles, the internal signal of the material shows a general homogeneity of facies, which allows us to exclude the presence of internal fracture and/or lesion surfaces. Even profiles P6 and P7, carried out in correspondence of a supposed clay vein, do not show internal separation surfaces that would generate reflections of the electromagnetic waves.

## 4. Discussion

The UST results indicate that the marble is generally well preserved, except for certain areas. Specifically, there are notable issues in the neck region and in the legs, particularly around the knees. These areas exhibit signs of deterioration that warrant further attention.

On the other hand, the GPR investigations, despite the inherent challenges associated with 3D modeling, reveal a consistent pattern in the electromagnetic properties of the marble. This consistency suggests that there are no significant internal anomalies within the marble structure.

**Figure 11:**
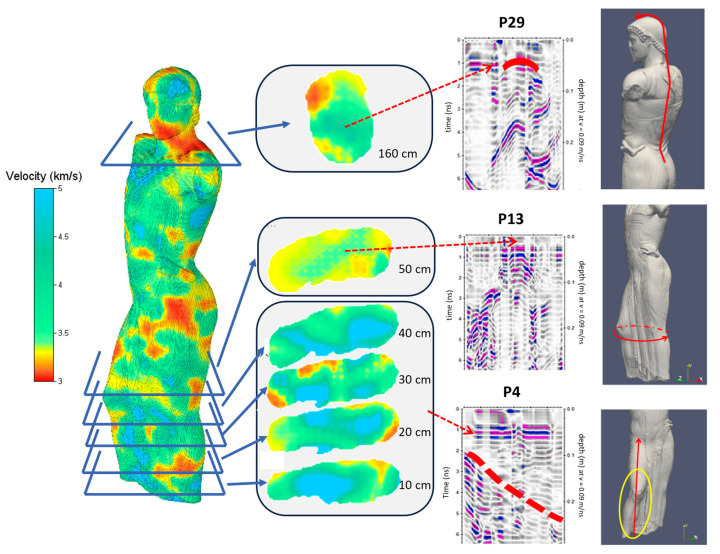
Joint interpretation of GPR and UST results. The red dashed arrows highlight the correspondences between US anomalies and GPR reflections. In the GPR profiles, he reflection amplitudes are represented in a color scale from blue (maximum negative amplitude) to magenta (maximum positive amplitude); the red continuous lines indicate the traces of GPR profiles on the surface of the statue; the continuous red line in P29 highlight the reflection of a metal pin.

The joint interpretation of the results of the two techniques (Figure 11) also showed an area in the neck that is characterized by a high ultrasonic velocity and a reflection of the GPR profile (P29), which is probably attributable to the presence of a pin, which could have been inserted during a previous restoration intervention. The same could be said regarding the P13 profile. In the lower area of the statue, the P4 profile highlighted an internal reflection instead, which is in correspondence with the visible superficial lesion. The corresponding ultrasound tomography highlights a low-velocity zone that does not, however, extend into the central part of the statue. This observation suggests that the damage is superficial and does not compromise the overall structural integrity of the statue, but nevertheless, the lesion must continue to be monitored over time.

## 5. Conclusions

This case study has demonstrated the effectiveness of the combined use of GPR and UST techniques, promoting the adoption of innovative and advanced approaches in the field of artistic diagnostics. By combining the high-resolution imaging of UST with the deep penetration capabilities of GPR, a more complete and detailed understanding of the marble’s condition is achieved. This synergy allows for the identification of both surface and deep-seated defects. Indeed, the integrated analysis of data from both techniques has proven to be of fundamental importance for the diagnosis of the statue, as it has allowed for a more detailed and precise understanding of the internal conditions of the marble, identifying any hidden defects or damages and detecting metal pins. It has been shown that, thanks to the joint use of these techniques, it is possible to intervene promptly to prevent further deterioration, ensuring optimal conservation and restoration of artworks and their safe transportation. This is particularly valuable for planning restoration and conservation efforts and is essential for making informed decisions about the preservation and maintenance of marble artifacts and structures.

The use of the ultra-thin film allowed for the preservation of the marble surface during the execution of the US and GPR investigations. A valuable suggestion for future research is a comparative analysis of measurements on a marble sample with and without the protective film. This would help in understanding the impact of the protective film on the spectrum of ultrasonic and electromagnetic impulses.

While our proposed methodology primarily focuses on volumetric imaging, one aspect that is not sufficiently investigated is the detailed analysis of the deterioration of the very superficial part of the marble. This can be improved by incorporating high-resolution photogrammetry and 3D laser scanning to capture the detailed surface topography. These techniques allow for the precise mapping of surface wear, cracks, and other forms of deterioration. Furthermore, to analyze subsurface features with higher resolution, we suggest using multispectral and hyperspectral imaging. These methods utilize various wavelengths of light to reveal hidden layers and damage to the first millimeters that are not visible to the naked eye. Finally, another complementary technique can be X-ray Fluorescence (XRF), which is useful for identifying the elemental composition of the surface, helping to detect chemical changes and corrosion. By integrating these techniques, we can provide a comprehensive assessment of both the surface and subsurface conditions of heritage objects, thereby enhancing the overall conservation strategy.

## Figures and Tables

**Table 1:** For each of the 30 GPR profiles carried out in 2021, the direction and position relative to the statue is given.

N.	Direction	Side	Approx. Length (cm)	Description
1	vertical	lateral	78	left lateral/retro leg up to the hand (measured 78 cm)
2	vertical	lateral	80	left frontal leg up to the hand
3	vertical	front	75	frontal center, following the clothing fold discrepancy, up to mid-torso
4	vertical	front	75	right leg, passing over the knee up to mid-torso
5	vertical	lateral	80	right leg, lateral up to mid-torso
6	vertical	lateral	40	zoom: Right lateral leg, focusing on the lesion, up to the knee
7	vertical	front	140	zoom: Right frontal leg, focusing on the lesion, up to the knee
8			14	calibration profile using metal plate: width of the calf is 14 cm
9	vertical	front	70	from mid-torso up to the neck, left leg side
10	vertical	front	70	from mid-torso up to the neck, right leg side
11	vertical	lateral	80	from mid-torso to the arm, right leg side
12	vertical	front	50	from mid-torso to chest—up to the neck
13	horizontal	circular	85	just below the knees, starting from the left leg—passing behind—then in front, intersecting the clothing fold that creates a reflection in the profile
14	horizontal	circular	95	at the calf, starting from the left leg—passing behind—then in front, intersecting the clothing fold that creates a reflection in the profile
15	horizontal	circular	100	just below the thigh, starting from the left leg—passing behind—then returning to the front of the left thigh
16	vertical	back	95	left leg, from bottom to top, up to the gluteus (including)
17	vertical	back	60	starting above the gluteus, passing beyond the shoulder, and reaching the chest in the frontal part
18	vertical	back/lateral	115	right leg, up to the shoulder
19	horizontal	circular	110	at the ribs, starting from the left torso, passing behind to the frontal torso
20	horizontal	front	40	below the neck, from the left shoulder to the right shoulder
21	vertical	back	95	left leg up to the gluteus (including)
22	vertical	back	80	central up to mid-buttock
23	vertical	back	95	right leg up to the gluteus (including)
24	vertical	back	38	from mid-torso (above the gluteus) up to the shoulder, left side
25	vertical	back	45	from mid-torso (above the gluteus) up to the neck, central
26	vertical	back	45	from mid-torso (above the gluteus) up to the shoulder, right side
27	vertical	lateral	35	from mid-torso up to the armpit—right leg side
28	horizontal	back	55	from left shoulder to right shoulder
29	vertical	back	80	from mid-torso (above the gluteus) up to the head (including)
30	horizontal	back	60	starting below the left armpit towards the right armpit

## Data Availability

Data can be obtained upon request from the corresponding author.
